# Micellar Antibiotics of *Bacillus*

**DOI:** 10.3390/pharmaceutics13081296

**Published:** 2021-08-19

**Authors:** William T. Ferreira, Huynh A. Hong, Mateusz Hess, James R. G. Adams, Hannah Wood, Karolina Bakun, Sisareuth Tan, Loredana Baccigalupi, Enrico Ferrari, Alain Brisson, Ezio Ricca, María Teresa Rejas, Wilfried J. J. Meijer, Mikhail Soloviev, Simon M. Cutting

**Affiliations:** 1 Department of Biological Sciences, Royal Holloway University of London, Egham TW20 0EX, UK; wuaz082@live.rhul.ac.uk (W.T.F.); hong.huynh@rhul.ac.uk (H.A.H.); mateusz.hess@kcl.ac.uk (M.H.); ja01329@surrey.ac.uk (J.R.G.A.); mikhail.soloviev@rhul.ac.uk (M.S.); 2 SporeGen Ltd., London Bioscience Innovation Centre, 2 Royal College Street, London NW1 0NH, UK; h.wood@sporegen.com (H.W.); k.bakun@sporegen.com (K.B.); 3 Laboratoire d’Imagerie Moléculaire et Nano-Bio-Technologie, UMR-CBMN CNRS-Université de Bordeaux-IPB, 33607 Pessac, France; s.tan@u-bordeaux.fr (S.T.); alain.brisson@u-bordeaux.fr (A.B.); 4 Department of Molecular Medicine and Medical Biotechnology, Federico II University of Naples, 80126 Napoli, Italy; loredana.baccigalupi@unina.it; 5 School of Life Sciences, University of Lincoln, Lincoln LN6 7TS, UK; eferrari@lincoln.ac.uk; 6 Department of Biology, Federico II University of Naples, 80126 Napoli, Italy; ericca@unina.it; 7 Centro de Biología Molecular “Severo Ochoa” (CSIC-UAM), C. Nicolás Cabrera 1, Universidad Autónoma de Madrid, Canto Blanco, 28049 Madrid, Spain; mtrejas@cbm.csic.es (M.T.R.); wmeijer@cbm.csic.es (W.J.J.M.)

**Keywords:** *Bacillus velezensis*, biosurfactant, micelle, antimicrobial, drug delivery, chlorotetaine

## Abstract

Members of the *Bacillus* genus, particularly the “*Bacillus subtilis* group”, are known to produce amphipathic lipopeptides with biosurfactant activity. This includes the surfactins, fengycins and iturins that have been associated with antibacterial, antifungal, and anti-viral properties. We have screened a large collection of *Bacillus*, isolated from human, animal, estuarine water and soil samples and found that the most potent lipopeptide producers are members of the species *Bacillus velezensis*. *B. velezensis* lipopeptides exhibited anti-bacterial activity which was localised on the surface of both vegetative cells and spores. Interestingly, lipopeptide micelles (6–10 nm diameter) were detectable in strains exhibiting the highest levels of activity. Micelles were stable (heat and gastric stable) and shown to entrap other antimicrobials produced by the host bacterium (exampled here was the dipeptide antibiotic chlorotetaine). Commercially acquired lipopeptides did not exhibit similar levels of inhibitory activity and we suspect that micelle formation may relate to the particular isomeric forms produced by individual bacteria. Using naturally produced micelle formulations we demonstrated that they could entrap antimicrobial compounds (e.g., clindamycin, vancomycin and resveratrol). Micellar incorporation of antibiotics increased activity. *Bacillus* is a prolific producer of antimicrobials, and this phenomenon could be exploited naturally to augment antimicrobial activity. From an applied perspective, the ability to readily produce *Bacillus* micelles and formulate with drugs enables a possible strategy for enhanced drug delivery.

## 1. Introduction

Found typically in the saprophytic layers of soil *Bacillus* can produce endospores (spores) that survive indefinitely in a dormant form [1]. As spores, the bacterium can be disseminated easily in air and dust and form a major part of the aerobiome [2]. Consumption of plants, vegetables and other matter introduces spores into the gastrointestinal (GI)-tract where their intrinsic robustness enables passage across the gastric barrier. In the small intestine (jejunum and ileum), spores germinate and proliferate and in the large intestine they sporulate before being shed in the faeces [3]. In the GI tract, numbers of *Bacillus* are noticeably low (~10^4^/g faeces in humans), but they are readily found in most animals suggesting that *Bacillus* is a minor allochthonous commensal [4]. Survival in dual communities (environment and intestinal) potentially presents an additional challenge to bacteria compared to those inhabiting a single niche. Production of antimicrobials plays an important part in competing with other bacteria and it is perhaps not surprising then, that members of the genus *Bacillus* are prolific producers of antimicrobials devoting 4–5% of its genome exclusively for this purpose [5,6]. Most of these compounds are peptides produced ribosomally (bacteriocins, enzymes) or non-ribosomally (lipopeptides, siderophores, polyketides) and are typically of low MW (5–10 kDa) [7,8]. In this work, we have screened *Bacillus* species isolated from diverse sources focusing on two phenotypic properties, antimicrobial activity and biosurfactant activity where the latter is typically indicative of the lipopeptide antibiotics (surfactin, fengycins and iturins [9]). We found that most strains exhibiting the highest levels of these dual activities belonged to one species, *Bacillus velezensis*. Using different pathogens including *Clostridioides difficile* we show that these *Bacillus* lipopeptides play an important role in antibacterial activity, first, by forming detergent-like mixed micelles and second, by incorporating antibiotics produced by *Bacillus*. The ability to incorporate lipopeptides and antibiotics into a micellar complex maximises antibacterial activity and is likely to have been adopted by this bacterium to its advantage.

## 2. Materials and Methods

### 2.1. General Methods and Strains

Strains screened for antimicrobial activity were presumed to be *Bacillus* due to the heat resistant step (68 °C, 30 min) and aerobic culture used in their isolation. Isolates in our collection were previously recovered from human (*n* = 166), animal (cows (*n* = 137), pigs (*n* = 117), chickens (*n* = 139), estuarine water (*n* = 143) and soil samples (*n* = 132). A well diffusion assay was used to screen supernatants from *Bacillus* strains grown for 18 h in BHIB (brain heart infusion broth) at 37 °C. Using appropriate media, indicator strains were grown to the mid-logarithmic phase at 37 °C and spread onto agar plates. Next, 50 μL of *Bacillus* supernatant (filter-sterilised) was applied to pre-cut wells (5 mm diameter). After overnight incubation, zones of inhibition were measured. The resistance properties of culture supernatants were assessed using heat (oven, 90 °C, 15 min), solvents, SDS (0.1% *v*/*v*) and glutaraldehyde (0.1% *v*/*v*) by mixing (1:1) and incubation at RT with periodic vortexing. Pathogen growth kinetic experiments were conducted using mid-logarithmically growing cultures of the target strain, dividing the culture into two and to one flask adding 1:10 volume of a cell-free supernatant of the relevant *Bacillus* strain. *C. difficile* (CD630) was routinely grown in BHIS medium (brain heart infusion supplemented with yeast extract and cysteine) agar (37 g brain heart infusion, 5 g yeast extract and 1 g L-cysteine per litre). For determining the minimum inhibitory concentration (MIC) of *Bacillus* lipopeptides, the CD630 culture was grown in BHIS until the OD_600_ reached ~0.2, at which point SG277 SEC purified lipopeptides were added at different 2-fold concentrations, in the range 1000–0.5 µg/mL (as estimated by dry weight). The cultures were left to incubate anaerobically for 18 h (37 °C). The OD_600_ of the cultures were measured and the MIC was determined, with the endpoint being defined as the lowest concentration where the OD < 50% of the CD630 “media only” control.

### 2.2. Biosurfactant Activity

Biosurfactant activities of *Bacillus* supernatants were determined using an oil-displacement method using sunflower oil [10].

### 2.3. Microdilution Assay of C. difficile Inhibitory Activity

Plates were set up as follows, 216 μL of sterile BHIS was pipetted into the first row of a 96-well U-bottom microplate (Greiner Bio-One, Gloucestershire, UK) and 120 μL into each subsequent row. Next, 24 μL of the sample to be tested was pipetted into the first row (dilution factor of 1:10) and serially diluted in a 2-fold dilution series until the last row (1:1280 dilution factor) on the microplate. A media-only control was also applied into a single well of the first column and serially diluted. Subsequently, 12 μL of a 6 h CD630 indicator culture was pipetted into each well and the plate was incubated for 18 h at 37 °C in an anaerobic chamber. After incubation, the microplate contents were agitated on a rotary plate shaker at 200 rpm for 2 min after which the OD_600_ was measured using a microplate plate reader. Positive inhibitory activity was defined as an OD_600_ < 50% of the CD630 “media only” control.

### 2.4. Purification Procedures 

For ammonium sulphate (AmSO_4_) precipitation, filter-sterile supernatant was precipitated overnight with AmSO_4_ (4 °C) using saturated AmSO_4_ (Sigma-Aldrich, Dorset, UK) (Supplementary Figure S3) solution at a final concentration of 20% (*w*/*v*). Following centrifugation at 10,000× *g* for 15 min, the supernatant was removed, and the precipitate was resuspended in PBS (Sigma-Aldrich, Dorset, UK). To remove excess AmSO_4_ the filtrate was dialyzed using 3500 daltons MEMBRA-CEL dialysis tubing (Viskase, Chesterfield, UK) against PBS overnight at 4 °C.

For size-exclusion chromatography (SEC) analysis, 1.5 mL of PBS was added to 1 mL of the dialysed AmSO_4_ precipitate and 2.5 mL applied to a Superdex 200 column (L × I.D. 30 cm × 10 mm) and fractionated by SEC with PBS as the running buffer. Fractions were tested for activity against CD630 using the microdilution assay and positive fractions combined, dialyzed against PBS overnight at 4 °C and concentrated using a 10 kDa Vivaspin (Sartorius, St. Neots, UK) MWCO centrifugal concentrator to a final volume of 1 mL. Henceforth, this is referred to as the active SEC fraction.

The SEC “active” sample was separated by RP-HPLC using uBondapack Phenyl, 125 Å, 10 µm, 3.9 × 300 mm (Waters, Manchester, UK) column and Waters 600 Multisolvent Delivery system, comprising Waters 600E System Controller and Waters 600 Fluid Unit, and complemented with an ABI 757 Spectroflow absorbance detector (Kratos, Manchester, UK). The mobile phase components were Buffer A (0.5% (*v*/*v*) acetic acid in 60% (*v*/*v*) MeOH) and Buffer B (0.5% (*v*/*v*) acetic acid in 95% (*v*/*v*) MeOH). The fractions were injected in Buffer A and the products eluted (flow rate 0.5 mL min^−1^) with a linear gradient of Buffer B, developed from 0% to 100% (60 min). The elution pattern was monitored by determining absorbance at 220 nm, and resultant fractions were concentrated using an EZ-2 Genevac centrifugal evaporator (Genevac Ltd., Ipswich, UK) and then either tested for inhibitory activity against CD630 (by resuspension in dH_2_O) using a microdilution assay or identified by mass spectrometry (MALDI-TOF).

MALDI-TOF-MS analysis was performed on a Bruker Reflex III system (Bruker Daltonics, Billerica, MA, USA) equipped with a sealed nitrogen pulsed laser (337 nm, line-width 0.1 nm, 4 ns pulse duration, 300 microjoules rated pulse energy, average power 5 mW at 200 Hz). A working solution of matrix was prepared by mixing 60 µL of the alpha-cyano-4-hydroxycinnamic acid matrix stock solution in MeOH (MALDI-QUALITY^TM^, Agilent Technologies, Santa Clara, CA, USA), 30 μL of acetone and 1 μL of 1% (*v*/*v*) TFA in a glass vessel. Individual RP-HPLC fractions were mixed with the matrix solution and deposited onto a stainless steel MALDI plate. Mass spectra were acquired using the reflection mode. The instrument was calibrated to a mass accuracy of at least 30 ppm using C18 purified sets of proteolytic albumin peptides to cover the range of masses from ca. 500 *m*/*z* to ca. 2000 *m*/*z*) and using α-cyano-4-hydroxycinnamic acid (CHCA, monoisotopic mass 189.0426) and CHCA clusters to map the lower MW region of ca. 200 *m*/*z* to ca. 800 *m*/*z*. In addition to extensive MALDI-TOF-MS analyses, masses were also confirmed using Matrix-Less Nanostructured Assisted Laser Desorption Ionisation TOF-MS (NALDI-TOF-MS) using a Bruker Reflex III system. In all cases, the TOF-MS analysis was conducted in reflector mode and 500 laser shots were accumulated for each mass spectrum.

### 2.5. Dynamic Light Scattering (DLS) Analysis

Active SEC material (in 150 mM sodium phosphate buffer, pH 7.2) was cleared by centrifugation at 17,000× *g* for 1 h prior to analysis. Next, 100 µL of sample was transferred to a micro-cuvette, equilibrated at 25 °C and measured in triplicate using a Zetasizer Nano ZS (Malvern Panalytical, London, UK). Particle diameter was estimated from the Z-average size. Polydispersity index (PDI) and cumulant fit intercept (CFI) were obtained from cumulants fit analysis using Zetasizer software v7.11 (Malvern Panalytical, London, UK). Data points are the average of three measurements with error bars representing the standard error.

### 2.6. Bactericidal Activity of SG277 Micellar Material

*B. velezensis* SG277 (a human isolate, SporeGen Ltd., London, UK) was grown in BHIB for 18 h at 37 °C resulting in >90% spore formation. The culture was centrifuged, and the supernatant was retained. The pellet from 1 mL of culture (~5 × 10^8^ CFU) was washed 3-times with PBS and resuspended in 1 mL of a mid-logarithmic stage CD630 culture. Cultures were gently agitated to prevent sedimentation of *Bacillus* spores. The collected supernatant was added to the same culture at a dilution factor of 1/10 (*v*/*v*), AmSO_4_ precipitate at 1/320 (*v*/*v*), SEC active fraction at 1/160 (*v*/*v*) and the RP-HPLC purified surfactin at 1/20 (*v*/*v*). The dilutions were determined according to normalisation of activity as measured in a previously performed microdilution assay. dH_2_O was added to the CD630 culture as a negative control. Samples were taken hourly, serially diluted and plated on BHISS agar plates and incubated for 24 h at 37 °C at which point colonies were enumerated and CFU/mL determined.

### 2.7. Microfiltration Experiments

SG277 was grown for 18 h in BHIB and the culture supernatant was centrifuged two-times (8000× *g*, 15 min, RT). The salt concentration of the supernatant was adjusted to ~150 mM and pH 7.0 by adding NaH_2_PO_4_H_2_0 (5% *w*/*v*) followed by centrifugation (21,000× *g*, 3 h, RT) to remove any aggregates. The cleared supernatant was then filtered through a series of syringe filters and 100, 30, 10 and 5 kDa Vivapsin (Sartorius, St. Neots, UK) MWCO cut-off spin columns (6000× *g* for 10 min). Each filter was first equilibrated with sterile BHIB medium before use. After filtration, eluates were collected, and membranes were washed with 1 mL of methanol (100%) in order to extract entrapped material. Eluates and the methanol filtered wash were checked for activity against CD630 using a microdilution inhibition assay. The outcome of the assay was registered as the lowest dilution at which the growth of CD630 was suppressed.

### 2.8. Agar Plate Inhibition Assay

SG277 was grown in BHIB for 18 h at 37 °C resulting in >90% spores (data not shown). The culture was centrifuged, and the supernatant retained. The pellet was washed 3 times with PBS. Both pellet (suspended in PBS) and retained supernatant were heat treated (80 °C, 15 min) to kill residual cells. Resuspended pellet or supernatant was mixed in equal volume (0.25 mL) with a logarithmic culture of CD630. This was then added to 4.5 mL of 0.6% micropropagation agar (Apollo Scientific, Manchester, UK) and poured on BHIS agar plates and incubated anaerobically overnight at 37 °C. Inhibition was apparent compared against the turbid appearance of untreated CD630.

### 2.9. Combination of Micelles and Antibiotics

The lipopeptide content of 30× AmSO_4_ (see above) was estimated to be ~10 mg/mL. The 30× AmSO_4_ was dried with a Genevac EZ-2 SpeedVac evaporator (Genevac Ltd., Ipswich, UK) and the material resuspended in methanol to a final concentration of ~0.2 mg/mL. This solution was then mixed with stock solutions of vancomycin (0.2 mg/mL), clindamycin (0.2 mg/mL) or metronidazole (1 mg/mL) (Sigma-Aldrich, Dorset, UK), to achieve a 1:1 micelle:antibiotic ratio. Methanol was removed from the mixture using a SpeedVac and the pellet was suspended in 1 mL of sterile 1× PBS (pH 7.4), bringing the concentration of micelles to ~1 mg/mL. The solutions were vortexed (15 min, RT) and then centrifuged (4000× *g*) in a tabletop centrifuge at 37 °C for 3 h and kept overnight at 4 °C. After overnight storage, the solutions were centrifuged (1 h, 4000× *g*, RT) and filtered through a 1.2 µm filter in order to remove any potential aggregates. The flow-through was then filtered again through a 10 kDa Vivaspin MWCO (4000× *g*, 1.5 h). The starting solution, retentate (MeOH wash of the membrane) and flow-through were equilibrated to the same volume and tested against 630 Δ*erm* (an erythromycin-resistant derivative of CD630) using a microdilution inhibition assay.

### 2.10. Micellar Lipopeptide Interactions with Resveratrol

A total of 50 µg of resveratrol (RSV) was mixed with 500 µL of SG277 SEC purified material (containing ~0.5 mg of lipopeptides) or PBS only (pH 7.4) and incubated while shaking for 18 h at RT. The mixture was centrifuged (21,000× *g*, 20 min), the supernatant collected and vacuum-evaporated until dry. The pellets were resuspended in MeOH, and resveratrol was detected and quantified against standards using RP-HPLC as described above, with absorbance at 295 nm determined. Aqueous solubility of resveratrol was calculated as a percentage of the solubilised quantity in the PBS only sample. For resveratrol micellar incorporation experiments, the above-collected supernatant was applied to a 10 kDa MWCO membrane, centrifuged (6000× *g*, 2 h) and the resulting eluate (<10 kDa) was removed. The retentate (>10 kDa), including a MeOH membrane wash, was also collected. The eluate and retentate solutions were vacuum evaporated until dry, resuspended in proportional volumes of MeOH, and resveratrol was detected and quantified against standards using RP-HPLC. The proportion of resveratrol in the eluate or retentate was calculated as a proportion of the total amount.

### 2.11. Cryogenic Electron Microscopy (Cryo-EM)

A 4 μL aliquot was placed onto an EM grid coated with a perforated carbon film. Excess liquid was removed with filter paper, and grids were plunge-frozen into liquid ethane cooled by liquid nitrogen using a Leica EMCPC cryo-chamber (Leica Microsystems, Milton Keynes, UK). For Cryo-EM observation, grids were mounted onto a Gatan 626 cryoholder (Gatan Inc., Pleasanton, CA, USA) and transferred to a Tecnai F20 microscope (ThermoFisher, Waltham, MA, USA) operated at 200 kV. Images were captured with an Eagle 2k CCD camera (FEI, Hillsboro, OR, USA).

### 2.12. Visualization by Transmission Electron Microscopy (TEM)

Samples (concentrated AmSO_4_ precipitated material) were adsorbed for 3 min to glow-discharged collodion/carbon-coated copper grids. Grids were then washed twice with bi-distilled water, negatively stained for 50 s with 2% uranyl acetate in water and air-dried before visualization under a JEM1010 transmission electron microscope (JEOL, Tokyo, Japan). Images were acquired with a TVIPS F416 CMOS camera (TVIPS, Gauting, Germany).

### 2.13. Biofilms

Biofilms were prepared on either DSM or S7 medium as described [11]. For SEM analysis, aluminium foils were placed into the wells of 12-well plates. The bacterial cultures were added (at an OD600 of 0.1) and incubated at 37 °C for 48–72 h under static growth conditions. After culturing, the biofilms were fixed with 2.5% glutaraldehyde (*v*/*v*) overnight at 5 °C, post-fixed with 1% osmium tetroxide (*w*/*v*) for 1.5 h at RT, dehydrated with ethanol, air-dried overnight and finally mounted on aluminium stubs. Samples were then washed with PBS, fixed and dehydrated. The dehydrated samples were coated with a gold layer and observed under a Scanning Electron Microscope (SEM) (FEI QUANTA 200, FEI company, Hillsboro, OR, USA) in high vacuum mode.

## 3. Results

### 3.1. B. velezensis Is a Prolific Producer of Antimicrobial Activity

Cell-free supernatants from over 800 *Bacillus* strains isolated from a variety of sources were screened for antimicrobial activity against a panel of Gram-positive (*Staphylococcus aureus, Staphylococcus hominis, Bacillus cereus, C. difficile* and *Listeria monocytogenes*) and Gram-negative (*Escherichia coli, Salmonella enterica* and *Klebsiella aerogenes*) pathogens using a well-diffusion assay. Approximately 80% of strains showed some level of inhibitory activity to at least one pathogen. Thirty-six strains that exhibited the highest levels of inhibitory activity were shortlisted and their species assigned using analysis of the *gyrA* and/or 16S rRNA genes (Supplementary Table S2). In each case, extracellular inhibitory activity was found to be resistant to heat (90 °C, 15 min) and apart from one (SG836) all strains carried strong biosurfactant activity in the cell-free culture supernatants. Except for four strains (SG215, SG655, SG836 and SG224), the remainder (*n* = 32) belonged to the “*B. subtilis* group” [12]. Most interesting was that ~80% of the isolated strains were *B. velezensis* (*n* = 24) or its close relative *B. amyloliquefaciens* (*n* = 2), which are known to typically exhibit biosurfactant properties due to the synthesis of lipopeptides including the surfactins, fengycins and iturins [13,14].

**Figure 1:**
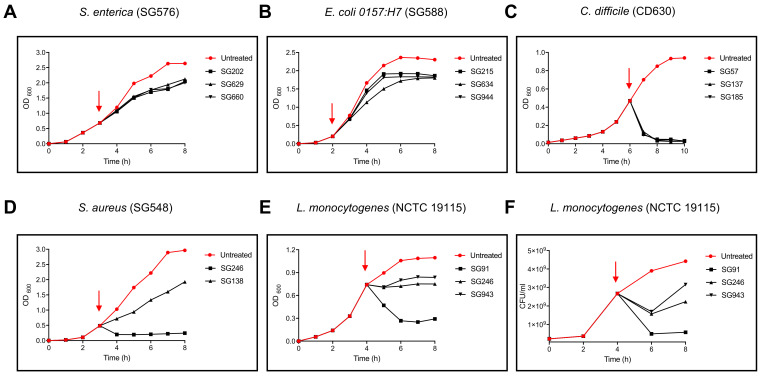
Pathogen growth kinetics in the presence of active components. Cell-free supernatants from *Bacillus* strain cultures (SGx) were added (1:10 *v*/*v*, indicated by red arrow) to mid-logarithmic cultures of Gram-negative and Gram-positive pathogens (**A**–**F**). (**A**) SG202, SG629 and SG660 supernatant was added (at time indicated by red arrow) to *S. enterica* (SG576); (**B**) SG215, SG634 and SG944 supernatant was added (at time indicated by red arrow) to *E. coli 0157:H7* (SG588), (**C**) SG57, SG137 and SG185 supernatant was added (at time indicated by red arrow) to *C. difficile* (CD630); (**D**) SG246 and SG138 supernatant was added (at time indicated by red arrow) to *S. aureus* (SG548); (**E**) SG91, SG246 and SG943 supernatant was added (at time indicated by red arrow) to *L. monocytogenes* (NCTC 19115); (**F**) SG91, SG246 and SG943 supernatant was added (at time indicated by red arrow) to *L. monocytogenes* (NCTC 19115). Growth (OD_600_) was monitored in cultures with or without (untreated) addition of supernatants. For *L. monocytogenes* growth by OD_600_ and by determination of CFU/mL.

Further analysis revealed that the nature of antibacterial activity varied considerably between strains with bacteriostatic, bactericidal as well as bacteriolytic activity (Figure 1, Supplementary Figure S1 and Supplementary Table S2). Bacteriostatic activity was observed against all Gram-negative pathogens while a broader range of activities (bacteriostatic, bactericidal or bacteriolytic) was evident against Gram-positives.

### 3.2. Characterisation of Inhibitory Activity

**Figure 2:**
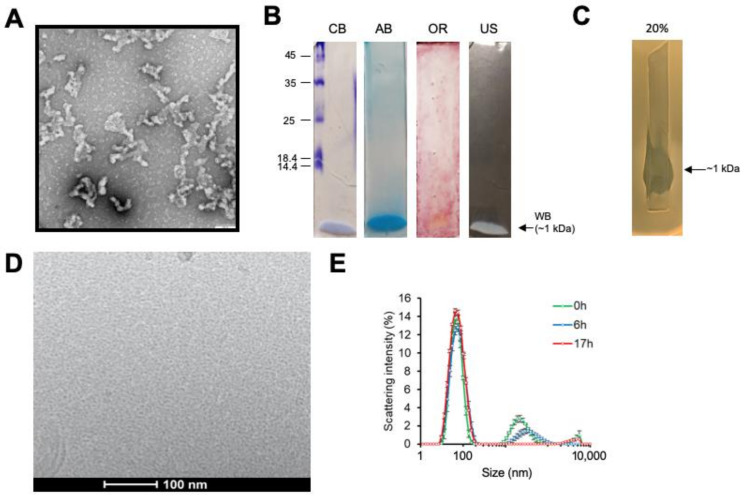
Activity corresponds to low MW species. (**A**) TEM analysis of AmSO_4_ precipitated material (20%), scale bar = 100 nm. (**B**) Staining of the 20% AmSO_4_ material run on 12.5% SDS-PAGE gels showed staining with Coomassie blue (CB) and Alcian blue (AB) but no detectable staining with oil-red (OR). Unstained gels (US) also showed clearly a 1 kDa unstained white band (WB). (**C**) The unstained gel lane was excised, SDS-eluted and the lane placed on BHIS agar and then overlaid with BHIS soft (0.6%) agar containing live CD630 cells. Following overnight growth under anaerobic conditions only the region corresponding to the 1 kDa (WB) band showed inhibition of CD630 growth. (**D**) Cryo-EM analysis of SEC material. The majority of the field is occupied with small (<10 nm) micelles appearing as granular-like objects. (**E**) DLS analysis of size distribution of the SG277 SEC fraction taken at different times following dilution in 150 mM sodium phosphate buffer at pH 7.2 shows the stability of the micelles over 17 h. Data points are the average of three measurements with error bars representing the standard error.

The abundance of *B. velezensis* strains with both heat-resistant inhibitory activity and biosurfactant properties might indicate a common mechanism of action. We focused on one pathogen, *C. difficile* strain 630 (CD630; ribotype 012, toxA^+^ toxB^+^), and the most potent *B. velezensis* strains (SG57, SG137, SG185, SG277 and SG297) for further analysis using a microdilution assay to quantify inhibitory activity. All strains were genome sequenced and confirmed as *B. velezensis* having ANI (average nucleotide identity) scores of >95% when compared to *B. velezensis* type strain ATR2 (Supplementary Table S1). SG277 and SG297 had inhibitory activities of 1:160 against CD630 while the remaining three strains had activities of 1:80. Activity in the culture supernatants exhibited complete resistance to heat (100 °C, 30 min), solvents (toluene, acetone and chloroform), SDS (0.1% *v*/*v*) and glutaraldehyde (1% *v*/*v*). Using centrifugal concentrators of different molecular weight cut-offs (MWCO) we determined that the antibacterial activity present in SG277 cell-free supernatants was present in two fractions, 30–100 kDa and >100 kDa suggesting the presence of a partially labile complex. In addition, we verified that equivalent levels of inhibition were present against our collection of over thirty *C. difficile* ribotypes (rt) including those known to be hypervirulent (rt 027) and zoonotic (rt 078) (data not shown). Ammonium sulphate (AmSO_4_) was also used for the precipitation of active material in the cell-free supernatant. Empirically, we found that a 20% (*w*/*v*) cut precipitated material that retained both inhibitory activity against CD630 (activity of 1:2560 in a 30× concentrate as measured using a microdilution assay) and biosurfactant activity. Transmission electron microscopy (TEM) of this precipitated material again revealed the presence of large granular aggregates of approximately 20–50 nm in size (Figure 2A). SDS-PAGE analysis of the AmSO_4_ precipitated material revealed a low MW band of ~1 kDa that was weakly stained with Coomassie blue and Alcian blue but not with oil-red (Figure 2B). Interestingly, a band of equal size and migration to a similar position was also apparent in “unstained” gels as a white species (labelled WB in Figure 2B). When the unstained gel lane was excised, SDS eluted and then overlaid with soft agar containing CD630 cells, following overnight anaerobic incubation, growth was inhibited in the region corresponding to the white band after overnight anaerobic incubation of the plate (Figure 2C). The AmSO_4_-precipitated active material was further purified by size exclusion chromatography (SEC) and reversed-phase HPLC (RP-HPLC). Cryo-EM analysis of SEC-purified material that retained inhibitory activity against CD630 revealed an abundance of homogenous structures resembling circular micelles with a diameter of ~6–10 nm (Figure 2D). DLS analysis confirmed that the material was homogeneous with most particles presenting a size within the 6–10 nm range, together with a minority population having a size of 150–200 nm (Figure 2E). Size distribution of these particles in sodium phosphate buffer (pH 7.2) was stable over several hours, with the only change being a reduction in aggregates larger than 100 nm indicating sedimentation.

### 3.3. Inhibitory Activity Is Due to the Formation of Mixed Micellar Complexes

**Figure 3:**
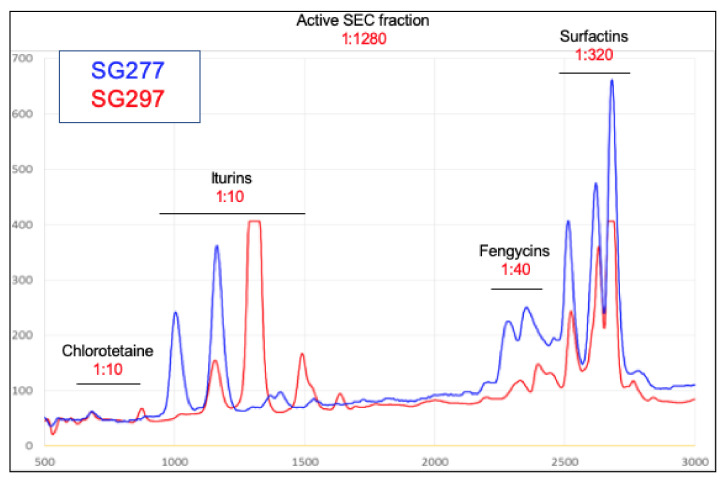
RP-HPLC separation and MALDI-TOF analysis of active components. RP-HPLC separation of the active SEC fraction from SG277 and SG297 using a gradient of 65–95% MeOH (+0.1% acetic acid). Peaks were analysed using MALDI-TOF and compounds are indicated in the figure. Initial inhibitory activity of the SEC fraction was 1:1280 and those in RP-HPLC fractions are indicated in red type.

SEC-purified material was separated by RP-HPLC, after which the individually collected fractions were tested for inhibitory activity against CD630 (Figure 3) and analysed by MALDI-TOF (Table 1). For active fractions derived from SG277 (and SG297) three classes of lipopeptide, surfactins, fengycins and iturins (iturin A and mycosubtilin) were readily detectable together with a dipeptide antibiotic chlorotetaine (MW ~300 Da; [15]). SEC purified material was determined to have an MIC of 4 µg/mL against *C. difficile*. Strains SG277 and SG297, which exhibited the highest levels of inhibitory activity against CD630, produced chlorotetaine, iturins, fengycins and surfactins. Strains that displayed lower inhibitory CD630 activity did not produce chlorotetaine (SG57, SG137 and SG185) or chlorotetaine and iturins (SG137 and SG185) (data not shown). Our analysis also revealed trace amounts of glycolipids including rhamnolipids and sophorolipids (data not shown) which may account for the Alcian blue staining (Figure 2B). However, we cannot rule out the possibility that this might also have arisen by contamination from the biofilm matrix (see below) which is typically rich in polyamines [16].

Using commercially acquired samples of surfactin (MW ~1 kDa), iturins (MW ~1 kDa) and fengycins (MW ~1.5 kDa) we were unable to demonstrate any significant level of inhibitory activity to CD630 even when all three lipopeptides were combined. It has been previously reported that surfactin C-15 (15 carbon fatty acid chain) is the homologue most active against cell membranes where the higher number of carbon atoms in the fatty acid chain in conjunction with the single negative charge in the peptide ring results in a more active compound [17,18]. Interestingly, contrary to the low proportion of surfactin C-15 present in the commercially acquired surfactin sample (Supplementary Figure S2A), samples prepared from the *Bacillus* strain contained higher levels of surfactin C-15 (Supplementary Figure S2D). In particular, a high level of inhibitory activity was observed only in the SG277 RP-HPLC fraction containing proportionally higher amounts of C-15 surfactin (Supplementary Figure S2B–D).

**Figure 4:**
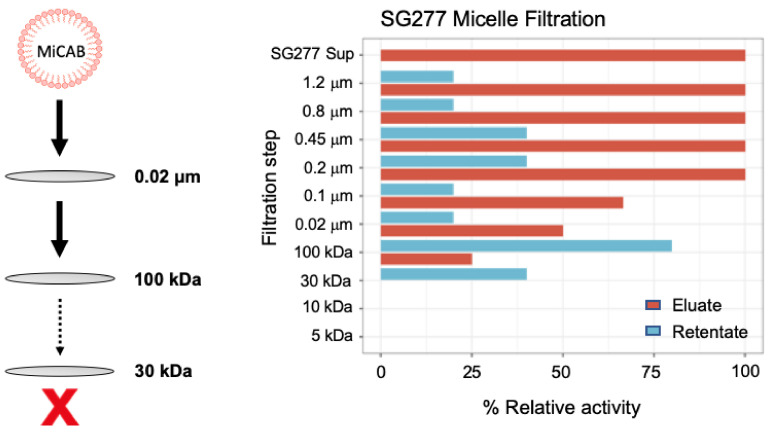
Activity is associated with micelles. Schematic diagram showing the *Bacillus* micelles and the step-wise filtration steps. The graph shows the resulting anti-CD630 activity in the eluate and retentate following filtration of an SG277 culture supernatant through a series of filters starting with 1.2 μm and ending with a 5 kDa MWCO membrane. Inhibitory activity to CD630 was determined using a microdilution assay and the data is the mean of 4 independent studies.

We next prepared a cell-free supernatant from an SG277 culture and passed this through a series of microfiltration membranes in a stepwise process. Starting with a 1.2 μM membrane, the supernatant was passed through and the activity in the eluate, as well as that retained on the filter, were determined (Figure 4). The eluate was then passed through further filters, at each step reducing the pore size, culminating in a 5 kDa MWCO membrane. This analysis showed that the 100 kDa MWCO membrane failed to allow passage of most of the inhibitory activity, with the remaining activity being retained on the subsequent 30 kDa filter. The 100 kDa MWCO membrane has a pore size of approximately 10 nm which is in close agreement with the predicted size of the SG277 micelles (Figure 2D–E) indicating the micelle size to be ~100 kDa. Some activity was retained using higher MWCO membranes, but we attribute this to the presence of larger aggregates that are apparent from DLS analysis (Figure 2D). This shows then that inhibitory activity is more likely associated with the micellar form rather than the individual components (all of which have MWs of <1.5 kDa). This is further supported by the high amount of anti-*C. difficile* activity observed when components are incorporated into mixed micelles (in the active SEC fraction), as opposed to the much lower levels of activity of individual components post RP-HPLC separation (Figure 3).

### 3.4. Genome Analysis

In strains with the highest activity against *C. difficile* strain CD630, surfactins, fengycins and iturins (both iturin A and mycosubtilin) were detectable by MS varying only by the length of their carbon chain. Trace levels of the lipopeptides Kurstakin [19] and Mojavensin A [20] were also detected. For strains with lesser activity (SG57, SG137 and SG185) only surfactins and fengycins were detectable by MS yet genomic analysis did reveal complete biosynthetic operons (*fenF* and *mycA-C*). Operons responsible for surfactins (*srfAA-D*) and fengycins (*yngL*, *fenA-E*) were present although in the case of the fengycins the order of cistrons within the operon did differ between strains (Supplementary Table S1). All strains carried three clusters of polyketide synthase genes common to *B. amyloliquefaciens* and responsible for the synthesis of difficidin (and its oxidised form oxydifficidin), bacillaene and macrolactins [5,21,22]. As indicated by the name, difficidin is a polyketide with activity against a number of bacteria including *C. difficile* [23] and although we were able to identify this antibiotic in SEC-purified material it was not present in the “active” RP-HPLC fractions. No gene or genes encoding chlorotetaine could be found but all strains carried an operon of five genes responsible for the closely related bacilysin (a dipeptide antibiotic commonly found in members of the *B. subtilis* group [24,25]).

### 3.5. Inhibitory Activity Associates with the Surface Layers of the Cell and Spore

**Figure 5:**
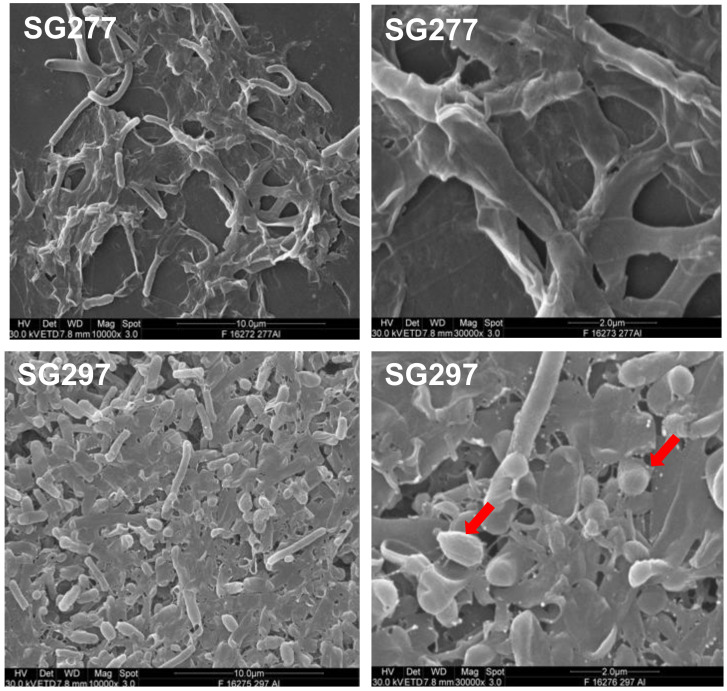
SG277 and SG297 biofilms. Biofilms developed in S7 medium and imaged using SEM. Red arrows highlight spores in the biofilm.

*B. velezensis* strains are, similar to those of *B. amyloliquefaciens*, highly mucoid resulting from the production of exopolysaccharides from the vegetative cell. We demonstrated that strains of this species could produce proficient biofilms in LB, DSM (Difco Sporulation Medium) as well and S7 medium (Figure 5).

**Figure 6:**
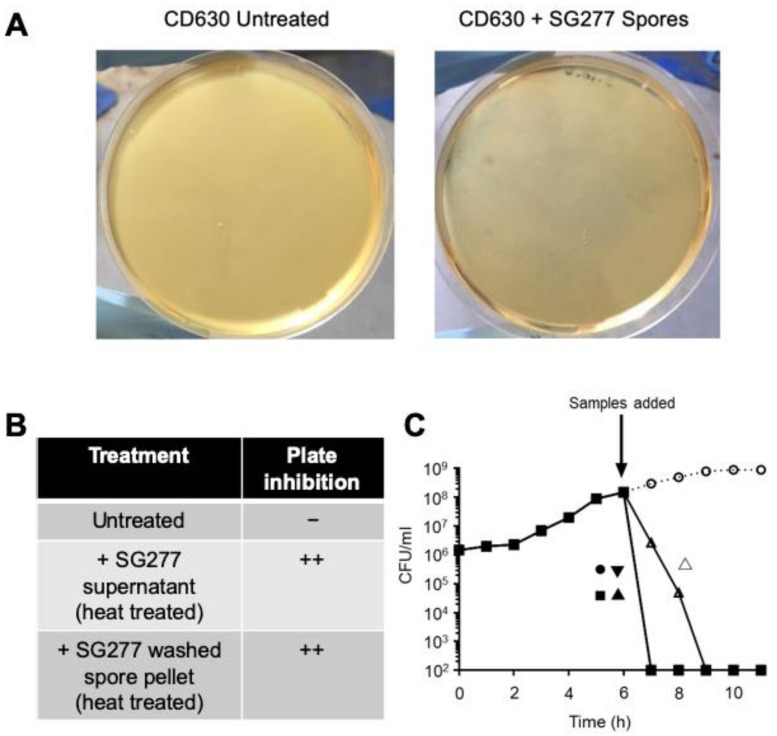
Inhibitory activity is associated with the surface of the cell and/or spore. (**A**,**B**) Heat-treated SG277 spores or supernatant were mixed in equal volumes with a logarithmic culture of CD630, added to 0.6% micropropagation agar and poured onto BHIS agar plates prior to anaerobic incubation (37 °C, 18 h). (**A**) Inhibition was apparent compared against the turbid appearance of untreated CD630. (**B**) Both the SG277 heat-treated supernatant and the spore pellet demonstrated agar plate inhibition of CD630 culture. (**C**) Fractions of “active” material obtained from *B. velezensis* SG277 were added to exponentially growing CD630 and viable bacteria (CFU/mL) were determined. Aqueous materials were: ●, cell-free supernatant; △, spores, consisting of purified spores of SG277; ■, AmSO_4_ precipitate; ▲, SEC active fraction; ▼, RP-HPLC purified surfactin; ○, dH_2_O (control). The activity of each fraction added was normalised prior to addition using a microdilution assay.

Spores were readily found in the biofilms together with vegetative cells in SG277 and other strains of *B. velezensis* when cultured for 18 h in brain heart infusion broth (BHIB). SG277 and other strains of *B. velezensis* demonstrated high levels of spore formation (>90%). To test a possible effect on CD630 cells, 18 h cultures of SG277 grown in BHIB (containing 90% spores and vegetative cells) were pelleted, washed repeatedly and then mixed with cells of CD630 (at the logarithmic phase of growth) and plated on soft agar (0.6%) plates. After anaerobic growth, the resulting plate exhibited complete inhibition when compared to those carrying CD630 alone (Figure 6A,B). Inhibition was also observed when a heat-treated, cell-free, SG277 supernatant was used. Under these conditions of anaerobiosis, strain SG277 was unable to grow, and the most probable explanation was that the inhibitory activity was intimately associated with the cell and/or spore surface. To further investigate, PBS-washed SG277 spores (~5 × 10^8^ CFU) were added to a CD630 culture, rapidly lysing the cells and demonstrating that active material could also associate with the spore surface (Figure 6C). Bacteriolysis was also observed with the cell-free supernatant, the 30× AmSO_4_ precipitated material, the SEC active fraction and RP-HPLC purified surfactin (Figure 6C). Methanol extraction of the spore surface followed by RP-HPLC analysis confirmed the presence of inhibitory lipopeptides (data not shown). 

### 3.6. Bacillus Micelles Can Incorporate Antimicrobials

**Figure 7:**
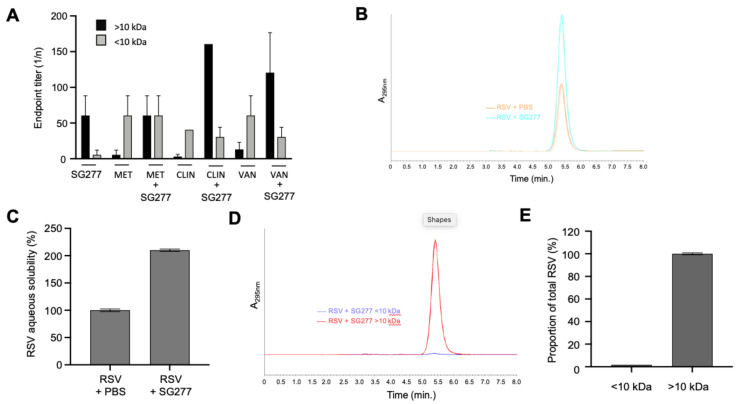
Micellar incorporation of antimicrobials. (**A**) SG277 AmSO_4_ material comprising micelles (*mic*) was mixed with either metronidazole (MET), clindamycin (CLIN) or vancomycin (VAN) and then filtered through a 10 kDa membrane. Inhibitory activity towards CD630 in the eluate (<10 kDa) was determined and compared to the starting solution. Antibiotics without the addition of micelles were taken through the same procedure for comparison. (**B**,**C**) SG277 SEC material or PBS were combined with resveratrol (RSV), incubated at RT and insoluble material was pelleted by centrifugation. (**B**) The supernatants containing solubilised resveratrol were analysed by RP-HPLC for resveratrol. (**C**) The absorbance was used to determine the aqueous solubility of resveratrol in SG277 SEC material as a proportion of resveratrol’s solubility in PBS. The experiment was performed 3-times. (**D**,**E**) SG277 SEC material was combined with resveratrol, incubated at RT and insoluble material was pelleted by centrifugation. (**D**) The supernatant was then filtered through a 10 kDa membrane and the eluate (<10 kDa) and retentate (>10 kDa) were analysed by RP-HPLC for resveratrol. (**E**) The absorbance was used to calculate the proportion of RSV in the eluate (<10 kDa) and retentate (>10 kDa). The experiment was performed 3-times.

Lipopeptides are known to form micelles but the discovery of chlorotetaine in the active material from some strains (SG277 and SG297) was intriguing since the inhibitory activity was greater with this antibiotic than without. Synthetically produced micelles have been used for intracellular drug delivery [26,27] and we speculated that this phenomenon could be used by *Bacillus* to enhance and concentrate antibacterial activity. To address this, we prepared an AmSO_4_ precipitate of an SG277 cell-free supernatant and mixed it with each of three antibiotics (clindamycin, vancomycin or metronidazole) that are known to inhibit the growth of *C. difficile*. Following incubation, the mixture was passed through a 10 kDa MWCO membrane and the activity towards CD630 was determined in the eluate and retentate (Figure 7A). SG277 AmSO_4_ material diluted to a concentration of ~0.2 mg/mL carried a basal level of CD630 inhibitory activity (1:40) that was unable to pass through the membrane due to its size (~100 kDa.) but was retained in the retentate. For each of the three antibiotics essentially all inhibitory activity passed through the membrane as expected. For clindamycin and vancomycin, residual levels were retained possibly resulting from binding to the filtration membrane. Interestingly, the inhibitory activity to CD630 of the micelle fraction (i.e., the fraction unable to pass through the 10 kDa membrane) was substantially increased when it was mixed with either clindamycin (3-fold increase) or vancomycin (2-fold increase). This increase in activity most probably results from the association of these antibiotics with the lipopeptide SG277 micelles. Lipopeptide micelles from *Bacillus* carry a hydrophobic interior surrounded by an anionic surface [28,29]. At physiological pH, as used here, clindamycin and vancomycin are positively charged [30,31] while metronidazole is neutral [32] possibly explaining the failure of metronidazole to interact with the micelles.

Biosurfactants are frequently used to aid in the solubilisation of hydrophobic biomolecules [33], and so we asked whether SG277 micelles could perform this function. SG277 SEC material or PBS only was saturated with resveratrol, an antimicrobial polyphenolic antioxidant of clinical interest [34,35]. After incubation, the mixture was centrifuged, and the supernatant was analysed by RP-HPLC for solubilised resveratrol (Figure 7B). The presence of SG277 micelles increased the aqueous solubility of resveratrol 2-fold (Figure 7C). This increase was achieved under the experimental conditions reported, and it is probable that further study may yield optimised conditions for enhancing resveratrol solubility using SG277 micelles. Polymeric micelles have been shown to enhance the bioavailability of drugs by increasing solubility [36], and so the effect shown here illustrates the potential of *Bacillus* lipopeptides to increase solubility and, therefore, the bioavailability of hydrophobic antimicrobials, including resveratrol.

Lipopeptide biosurfactants associate with other molecules via electrostatic bonding and hydrophobic interactions [37]. Hydrophobic, poorly water-soluble molecules typically interact with the interior core of micelles, as this is where the hydrophobic, lipophilic tail segments are located [38]. It follows then that in addition to increasing the solubility of resveratrol, *Bacillus* lipopeptides may be incorporating the hydrophobic antimicrobial into the complex. To address this, SG277 SEC material was incubated with resveratrol and following centrifugation, the supernatant containing soluble SG277 micelles + resveratrol was passed through a 10 kDa MWCO membrane. The eluate (<10 kDa) and the retentate (>10 kDa) were analysed by RP-HPLC for the presence of LMW resveratrol (228 Da) (Figure 7D). Interestingly, the lipopeptide micelles retained over 99% of the LMW resveratrol and prevented it from passing through the 10 kDa membrane demonstrating that the antimicrobial had been incorporated into the micelle (Figure 7E).

## 4. Discussion

We have shown here that *B. velezensis* is a prolific producer of antimicrobial activity. *B. velezensis* is a recently reported heterotypic synonym of *B. amyloliquefaciens* [12,39] and shares with it several similarities, e.g., mucoid colonies and production of amylase. The description of this new species also makes it likely that many strains previously reported as *B. amyloliquefaciens* are in fact *B. velezensis*. As with other members of the *B. subtilis* group, *B. amyloliquefaciens* and *B. velezensis* produce lipopeptide antibiotics including surfactins, iturins and fengycins as well as the polyketide antibiotics, difficidin, bacillaene and macrolactins [40,41]. The lipopeptides have been well characterised in *Bacillus* and are known for their antibacterial [8], anti-fungal [14] and anti-viral [42,43] activity. While antimicrobial and biosurfactant activity associated with *Bacillus* is not unexpected, a direct link between the two is.

A key finding in this work is that lipopeptide micelles incorporate antibiotics, in this case, chlorotetaine. Numerous studies have reported on *Bacillus* strains with antimicrobial activity, but effort has focused on the purification of individual components rather than the whole. For example, to illustrate the efficacy of difficidin and bacilysin from *B. amyloliquefaciens* FZB42, analysis was made using isogenic mutants devoid of the lipopeptides surfactin, fengycin and iturins [44]. Commonly used purification procedures will break down micelles and bias effort towards the low MW species (<2 kDa). As we have shown here, the activity of individual RP-HPLC fractions carry inhibitory activity, but this is far less than when incorporated into a mixed micelle. Accordingly, by focusing on low MW species the true activity, conferred by the micellar formulation, will most likely have been overlooked.

Micelles will form only when the concentration of individual lipopeptide monomers has exceeded the critical micelle concentration (cmc) and in this form, biosurfactant properties arise [29]. In micellar form, lipopeptides efficiently disrupt not only membranes but also proteins [45]. This activity is focused on the cell and/or spore surface and probably also provides a competitive advantage in multicellular communities for enhancing motility, swarming activity and colonisation [46]. As well as entrapping and concentrating activity, the micelle may also provide a mechanism to enhance the stability of individual antimicrobials. For example, purified difficidin has been shown to have poor stability at different pHs and is readily oxidised [47].

Although lipopeptide micelles have been extensively studied at a biophysical level, less is known about those produced *in vivo* by *Bacillus* (and it should be emphasized that we have only examined this phenomenon in *B. velezensis* strains). We have found that the composition of lipopeptides within micelles does differ between strains and evidence is presented here that (a) a full cohort of surfactins, fengycins and iturins have greater functionality, and (b) the length (C_-x_) of the hydrophobic fatty acid chain is important. Based on the results obtained, it is tempting to speculate that micelle formation may be additive, that is, micelles form from a pool of available amphiphilic molecules and the final composition of micelles will reflect the relative abundance of these lipopeptides at the cell surface. This might explain why in some strains we have examined (e.g., SG137 and SG185) iturins were not detected in the micelles. We also cannot rule out the inclusion of glycolipids (notably rhamnolipid) and in other work, a *B. subtilis* strain has been reported that carries biosurfactant activity composed of two lipopeptides (surfactin and fengycin) and glycolipids (rhamnolipids) [48].

Interaction of micellar aggregates with the hydrophilic cell surface might be expected but the spore surface is typically hydrophobic [49]. Possibly, the spores are themselves coated in bacterial exopolysaccharides derived from the culture medium and our analysis of biofilms (Figure 5) shows spores embedded in the biofilm matrix that supports this assumption. Spores have also been shown to efficiently bind lipopolysaccharides [49] and the spore may provide a suitable platform for the aggregation of lipopeptides particularly in biofilm matrices. Clearly, in multicellular communities, the accumulation of entrapped antimicrobials on the spore or cell surface may offer a competitive advantage.

Strains with the greatest activity to *C. difficile* produced micelles containing chlorotetaine. Chlorotetaine is a little-studied dipeptide antibiotic that is closely related to bacilysin [50,51]. Bacilysin is present in most members of the *B. subtilis* group and all strains of *B. velezensis* examined here carried a bacilysin operon yet no operon that might encode chlorotetaine was found. A single halogenation step is required to convert bacilysin to chlorotetaine [52] and it is most likely, therefore, that bacilysin is modified to chlorotetaine in strains SG277 and SG297 either using a halogenase or alternatively by exogenous chlorine.

Probably one of the primary roles of lipopeptides may be to form micelles enabling the capture of other *Bacillus*-produced antimicrobials. We would then expect that for strains exhibiting inhibitory activity specific to other bacteria that other micellar-antibiotic formulations might exist. It should be noted that more focused and targeted activity has also been reported for *Bacillus* lipopeptides. For example, with inhibitory activity to *S. aureus.* Here, *B. subtilis* fengycins inhibit the Agr (accessory gene regulator) quorum-sensing system by competitive inhibition with the Agr auto-inducing peptide [53]. In our work, we observed both bacteriostatic and bactericidal activity against *S. aureus* suggesting different mechanisms of action which is intriguing. We also found that for Gram-negatives only bacteriostatic activity was found while for the Gram-positive pathogens a range of activities (bacteriostatic, bactericidal and bacteriolytic) were evident. The cell envelopes of Gram-positives and Gram-negatives obviously differ with the former being more susceptible to detergents [54]. Since we only examined the precise mechanism of action to *C. difficile*, we cannot exclude the possibility that for the other pathogens alternative mechanisms exist.

It seems logical to propose that micelles may have been exploited by *Bacillus* to improve antimicrobial performance. Recently, a comparable phenomenon has been observed in *Clostridium scindens,* whereby micelle-forming secondary bile acids were shown to combine with tryptophan-derived antibiotics, thereby increasing the activity against the intestinal pathogen *C. difficile* [55]. It should be emphasised that individual lipopeptides, as well as chlorotetaine, do show activity to *C. difficile,* but this is less than when combined (Figure 3). One apparent anomaly then is our observation that when fractionated by SDS-PAGE an active species of 1 kDa showed *in situ* activity to CD630 (Figure 2B). We cannot correlate the level of this activity to those determined through a microdilution assay, but one possibility is that the lipopeptides re-form into high molecular weight micelles following the removal of SDS, which is able to disrupt intermolecular bonding [56].

One surprising observation made here is the presence of large aggregates 20–50 nm in size. These granular clusters were primarily larger than the 10 nm micelles observed by cryo-EM and potentially represent supermicelles formed by supramolecular assembly [57]. DLS analysis showed evidence of a large aggregate size and possibly these aggregates might be easily entrapped in the exopolysaccharide layers of the cell envelope.

In summary, we have demonstrated the superior antimicrobial activity of a naturally occurring mixture of lipopeptides, as compared with a single lipopeptide species. Solubilisation of drugs using synthetic micelles [58] is a promising therapeutic approach now being developed and so it is useful to consider whether *Bacillus* lipopeptides could be exploited for nanoparticle micelle drug delivery.

## Figures and Tables

**Table 1:** Components identified during MALDI-TOF analysis for peaks collected during RP-HPLC separation of the SG277 active SEC fraction.

Compounds	SG277/SG297 Identities
Chlorotetaine	Chlorotetaine (^35^Cl) (^37^Cl)
	Hydroxychlorotetaine (^35^Cl) (^37^Cl)
Iturins	C_12-17_ Iturin
	C_11-17_ Bacillomycin F
	C_11-17_ Mycosubtilin
Fengycins	C_15-19_ Fengycin A
	C_13-17_ Fengycin B
	C_17_ Fengycin C
	C_16_ Fengycin D
	C_17_ Fengycin E
Surfactins	C_12-17_ Surfactin A
	C_13-18_ Surfactin B
	C_12-17_ Surfactin C

## Data Availability

The data presented in this study are available from the corresponding author upon reasonable request.

## Supplementary Materials

The following are available online at https://www.mdpi.com/article/10.3390/pharmaceutics13081296/s1, Figure S1: Pathogen growth kinetics using CFU counts, Figure S2: MALDI-TOF analysis of commercial vs *Bacillus* surfactins, Figure S3: Purification steps, Table S1: Genome analysis of lipopeptide, polyketide and bacilysin genes present in *B. velezensis* “active” strains., Table S2: Antimicrobial screening.
