# Supplementary material for: Micellar Antibiotics of Bacillus
Source: Pharmaceutics. 2021 Aug 19;13(8):1296. doi: 10.3390/pharmaceutics13081296 (PMC8399155; doi:10.3390/pharmaceutics13081296)
Supplement: Supplementary file 1 [file pharmaceutics-13-01296-s001.zip › pharmaceutics-1334146-supplementary.pdf]

pharmaceutics

MDPI

Supplementary Materials: Micellar Antibiotics of *Bacillus*

William T. Ferreira, Huynh A. Hong, Mateusz Hess, James R. G. Adams, Hannah Wood, Karolina Bakun, Sisareuth Tan, Loredana Baccigalupi, Enrico Ferrari, Alain Brisson, Ezio Ricca, María Teresa Rejas, Wilfried J.J. Meijer, Mikhail Soloviev and Simon M. Cutting

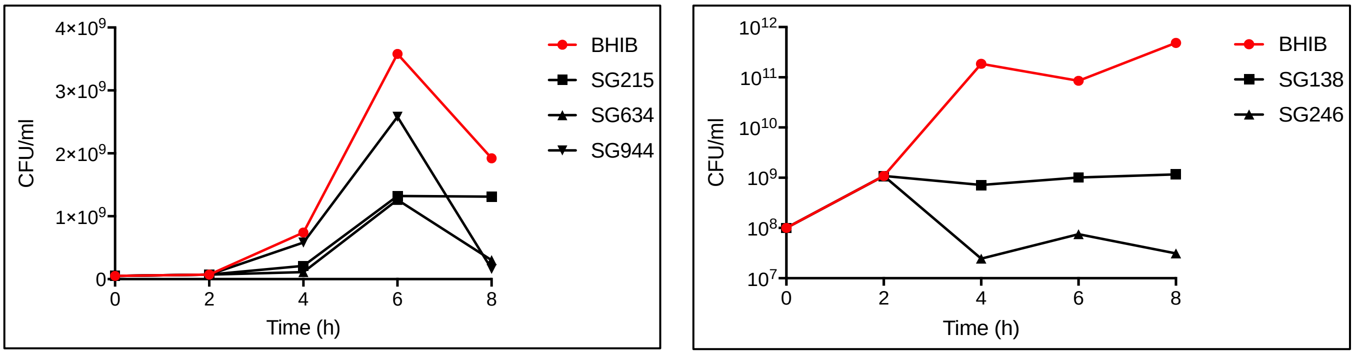

(**A**) E. coli 0157:H7 (SG588) (**B**) S. aureus (SG548)

Figure S1. Pathogen growth kinetics using CFU counts. Cell free supernatants from *Bacillus* strain cultures (SGx) were added (1:10 *v*/*v*, indicated by red arrow) to mid-logarithmic cultures of Gram negative and Gram-positive pathogens. Growth was monitored by determination of CFU/ml in cultures with or without (untreated) addition of supernatants.

Table S1. Genome analysis of lipopeptide, polyketide and bacilysin genes present in *B. velezensis* 'active' strains. Operons identified from genome sequencing of *B. velezensis* strains with activity to *C. difficile*. Activities determined to CD630 using a microdilution assay are shown as the dilution factor. ANI values (two-way) compared to *B. velezensis* (NCBI: NZ_CP018133.1) are given as a % and were determined using the Enveomics program [1]. The order of ORFs in the fengycin operon differs between strains as indicated.

Antimicrobial	SG57	SG137	SG185	SG277	SG297
a-CD630 activity	1:80	1:80	1:80	1:160	1:160
ANI value	97.25	98.00	97.99	97.59	98.90
surfactin	*srfAA-D*	*srfAA-D*	*srfAA-D*	*srfAA-D*	*srfAA-D*
**fengycin**	*yng-fenBAEDC*	*yng-fenBAEDC*	*yng-fenBAEDC*	*yng-fenED-//-CBA*	*yng-EDCBA*
iturins	*fenF, mycA-C*	*fenF, mycA-C*	*fenF, mycA-C*	*fenF, mycA-C*	*fenF, mycA-C*
bacilysin	*bacA-E*	*bacA-E*	*bacA-E*	*bacA-E*	*bacA-E*
bacillaene *(pks1)*	*pksA-S*	*pksA-S*	*pksA-S*	*pksA-S*	*pksA-S*
macrolactin *(pks2)*	*mlnA-H*	*mlnA-H*	*mlnA-H*	*mlnA-H*	*mlnA-H*
difficidin *(pks3)*	*dfnA-O*	*dfnA-O*	*dfnA-O*	*dfnA-O*	*dfnA-O*

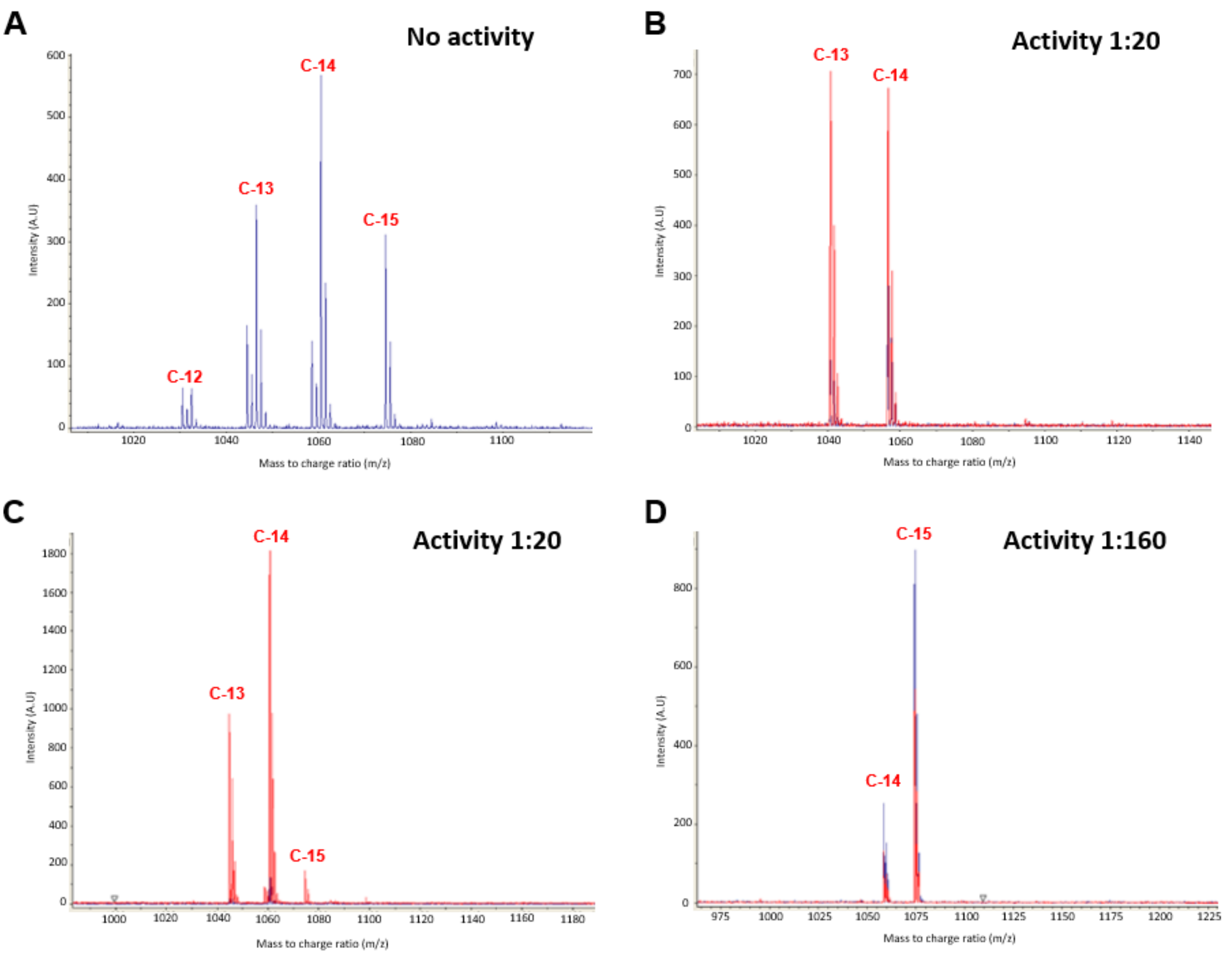

Figure S2. MALDI-TOF analysis of commercial vs *Bacillus* surfactins. MALDI-TOF analysis of commercially acquired surfactin (Sigma S3523) (**A**) and surfactin from three distinct 'active' fractions collected after RP-HPLC separation of the SG277 SEC material; active fraction exhibiting activity against CD630 of 1:20 (**B**), active fraction exhibiting activity against CD630 of 1:20 (**C**), active fraction exhibiting activity against CD630 of 1:160 (**D**). Activity against CD630 (1/dilution factor) and surfactin species are indicated in the figures. Activity was greatest in the fraction containing C-15 surfactin (**D**).

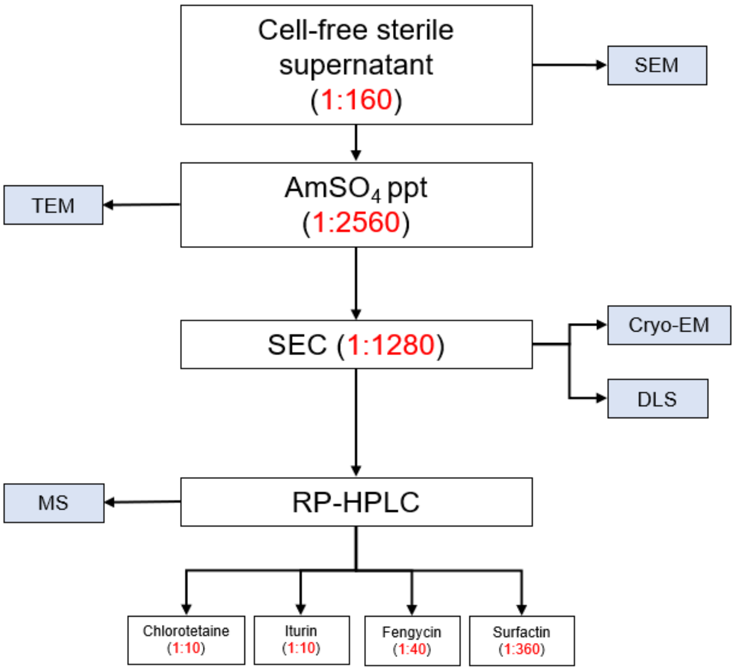

Figure S3. Purification steps. Flow diagram showing purification steps of culture supernatants and analyses performed. TEM: transmission electron microscopy, MS: MALDI-TOF analysis, DLS: dynamic light scattering, SEC: size exclusion chromatography, RP-HPLC: Reversed-phase high performance liquid chromatography. Activity against CD630 using a microdilution assay is indicated in brackets.

Table S2. Antimicrobial screening[a.]

Group[b]	Strain No.	Species[c]	Biosurfactant activity[d]	Heat resistance[e]	Gram-negative			Gram-positive				
					E. coli (0157:H7; 078:K80)	*Salmonella enterica (NCTC 13349:SG576)*	*K. aerogenes (NCTC 1006)*	*B. cereus (SG584)*	*C. difficile (630)*	*S. hominis (ATCC 27844)*	*S. aureus (SG548)*	*L. monocytogenes (NCTC 19115)*
"*B. subtilis*"	SG634	*B. velezensis*	+++	+	BS						BS	
	SG113	*B. velezensis*	+	+	BS							
	SG661	*B. velezensis*	+++	+	BS							
	SG630	*B. velezensis*	+	+			BS					
	SG184	*B. velezensis*	+++	+		BS					BS	
	SG545	*B. velezensis*	+	+								BC
	SG91	*B. velezensis*	++	+				BC		BS	BC	BC
	SG309	*B. velezensis*	+	+								BC
	SG541	*B. velezensis*	+	+				BC				
	SG944	*B. velezensis*		+	BS					BS		
	SG71	*B. velezensis*	+	+		BS				BS		
	SG660	*B. velezensis*	++	+		BS				BS		
	SG677	*B. velezensis*	+++	+							BS	
	SG57	*B. velezensis*	++	+					BL			
	SG137	*B. velezensis*	++	+					BL			
	SG185	*B. velezensis*	++	+					BL			
	SG277	*B. velezensis*	+++	+					BL			
	SG297	*B. velezensis*	+++	+					BL		BC	
	SG202	*B. velezensis*	+	+		BS					BC	
	SG943	*B. velezensis*		+							BS	BS
	SG633	*B. velezensis*	+	+							BC	
	SG629	*B. velezensis*	+	+		BS					BC	
	SG695	*B. velezensis*	+	+							BC	
	SG138	*B. velezensis*	++	+							BS	
	SG18	*B. amyloliquefaciens*	+	+	BS							
	SG246	*B. amyloliquefaciens*	+	+		BS					BC	BS
	SG531	*B. subtilis*	+	+				BS				
	SG308	*B. subtilis*	++	+		BS						
	SG281	*B. subtilis*	+	+		BS						
	SG691	*B. subtilis*	+++	+	BS							
	SG3	*B. subtilis*	++	+	BS							
	SG310	*B. mojavensis*	+++	+			BS					
Other	SG215	*B. infantis*	+	+	BS							
	SG836	*B. pseudomycoides*	-	+			BS					
	SG655	*Viridibacillus arvi*	++	+			BS					
	SG224	*Mesobacillus subterraneous*	++	+								BS

[a] cultures from strains were tested for inhibitory activity to a panel of Gram negative and Gram positive pathogens. Initial screening was by well diffusion and secondary screening using co-culture where activity is defined as BS, bacteriostatic, BC, bactericidal or BL, bacteriolytic. Strains were obtained from strain collections or were lab stocks.[b] operational group of related species, [2].[c] species assigned by *gyrA* sequence analysis [3] for members of the *B. subtilis* group and 16S rRNA [4,5] for remaining strains. [d] biosurfactant activity defined as zone of oil displacement; +, <10mm; ++. 10–20mm; +++, >20mm.[e] inhibitory activity present after heating of cell-free supernatant for 15 min. at 90°C.

References:

1. Rodriguez-R, L.M.; Konstantinidis, K.T. The enveomics collection: a toolbox for specialized analyses of microbial genomes and metagenomes. *PeerJPreprints* **2016**, e1900v1, doi:10.7287/peerj.preprints.1900v1.
2. Fan, B., Blom, J., Klenk, H.P., Borriss, R. *Bacillus amyloliquefaciens*, *Bacillus velezensis*, and *Bacillus siamensis* Form an "Operational Group *B. amyloliquefaciens*" within the *B. subtilis* Species Complex. *Front Microbiol.* **2017**, *8:22*, doi: 10.3389/fmicb.2017.00022.
3. Chun, J.; Bae, K.S. Phylogenetic analysis of *Bacillus subtilis* and related taxa based on partial *gyrA* gene sequences. Antonie Van Leeuwenhoek **2000**, *78*, 123–127, doi: 10.1023/A:1026555830014.
4. Green, D.H., Wakeley, P.R., Page, A., Barnes, A., Baccigalupi, L., Ricca, E., Cutting, S.M. Characterization of two *Bacillus* probiotics. *Appl. Environ. Microbiol.* **1999**, *65*, 4288–4291.

5. Hoa, N.T., Baccigalupi, L., Huxham, A., Smertenko, A., Van, P.H., Ammendola, S., Ricca, E., Cutting, A.S. Characterization of *Bacillus* species used for oral bacteriotherapy and bacterioprophylaxis of gastrointestinal disorders. *Appl. Environ. Microbiol.* **2000**, *66*, 5241–5247.
